# Predictors of lung function test severity and outcome in systemic sclerosis-associated interstitial lung disease

**DOI:** 10.1371/journal.pone.0181692

**Published:** 2017-08-01

**Authors:** Noémie Le Gouellec, Alain Duhamel, Thierry Perez, Anne-Lise Hachulla, Vincent Sobanski, Jean-Baptiste Faivre, Sandrine Morell-Dubois, Marc Lambert, Pierre-Yves Hatron, Eric Hachulla, Hélène Béhal, Regis Matran, David Launay, Martine Remy-Jardin

**Affiliations:** 1 Univ. Lille, U995, Lille Inflammation Research International Center (LIRIC), Lille, France; 2 Inserm, U995, Lille, France; 3 CHU Lille, département de médecine interne et immunologie clinique, Lille, France; 4 Centre national de référence maladies systémiques et auto-immunes rares (sclérodermie systémique), Lille, France; 5 UDSL, EA 2694, UFR Médecine, Lille, France; 6 CHRU Lille, Service d’Explorations Fonctionnelles Respiratoires, Lille, France; 7 CHRU Lille, Département d’Imagerie Thoracique, Lille, France; Keio University, JAPAN

## Abstract

Systemic sclerosis-related interstitial lung disease (SSc-ILD) is the leading cause of death in SSc. In this study, we aimed to describe the baseline severity and evolution of forced vital capacity (FVC) and diffusing capacity for carbon monoxide (DLCO) in patients with SSc-ILD and to assess the baseline clinical, biological and high-resolution CT scan (HRCT) predictors of this evolution. Baseline and serial FVC and DLCO were collected in 75 SSc-ILD patients followed during 6.4±4.2 years (n = 557 individual data). FVC and DLCO evolution was modelled using a linear mixed model with random effect. During follow-up, FVC was stable while DLCO significantly decreased (-1.5±0.3%/year (p<0.0001). Baseline NYHA functional class III/IV, extensive SSc-ILD on HRCT and DLCO<80% were associated with a lower baseline FVC. Absence of digital ulcers extensive SSc-ILD, and FVC<80% and were associated with a lower baseline DLCO. Presence or history of digital ulcers and presence of pulmonary hypertension at baseline or during follow-up were associated with a faster decline of DLCO overtime. Neither age, gender, subtype of SSc nor specificity of autoantibodies were associated with baseline severity or outcome of lung function tests. In this SSc-ILD population, FVC was therefore stable while DLCO significantly declined over time. ILD extension was associated with baseline FVC and DLCO but not with their evolution. Presence or history of digital ulcers and pulmonary hypertension were predictors of a faster decline of DLCO over time.

## Introduction

In systemic sclerosis (SSc), pulmonary complications, namely interstitial lung disease (ILD) and pulmonary hypertension (PH), are frequent and the leading cause of mortality in SSc patients [1–5]. Treating ILD is challenging because recent trials have shown no or an apparently modest benefit of immunosuppressants [6, 7] in patients with mostly stable ILD. However, predicting the outcome of ILD in SSc is important because treating patients whose lung function tests are worsening or expected to worsen is an important current strategy aimed at improving the benefit/risk ratio of immunosuppressants [8–10]. Yet, while it is proven that the more extensive the ILD at baseline, the worse the survival [11, 12], predicting lung function tests outcome (i.e. lung volumes and diffusing capacity for carbon monoxide [DLCO] measured during pulmonary function tests [PFTs]) in SSc-associated ILD (SSc-ILD) is still a matter of issue. Among the reasons for the discrepancies on predictors of PFTs evolution found in the literature, two seem important to highlight. Firstly, the SSc population can vary between studies. Some studies included patients from a tertiary centre for ILD [10], from randomized trials for ILD in SSc with inclusion of rather severe patients [6, 7] or from tertiary centres for SSc [12]. However, in many centres managing SSc patients, ILD is often mild and there is a paucity of data on the PFTs outcome in these milder SSc-ILD. Secondly, there are differences in the way worsening of PFTs is defined. In most studies, worsening was defined as a decrease of more than 10% of forced vital capacity (FVC) and/or more than 15% of DLCO during serial PFTs [10]. In other studies, serial changes of FVC used as a continuous variable or lung transplantation/oxygen requirement were chosen as an endpoint [11].

If one attempts to combine the findings of all these studies, the results are conflicting as some authors found that only diffuse SSc [13], only antitopoisomerase 1 [12], or mainly high-resolution computed tomography scan (HRCT) extension [10] was predictive of PFTs worsening in SSc-ILD.

Most of the studies used a “significant decline” of FVC and/or DLCO to define SSc-ILD worsening and to assess “time to decline” [10]. However, while these values are widely accepted, there are major issues. First, the time to decline is highly dependent on the intervals between PFTs. The optimal time interval for repetition of PFTs is not defined and probably varies between non-severe and more severe patients. Secondly, FVC or DLCO can spontaneously improve in SSc patients and patients fulfilling the criteria of “significant decline” at one PFT may not fulfill them in the following one. One way to be independent of the time interval between PFTs and to take account of the whole PFTs is to use a linear mixed model (LMM) integrating all serial PFTs [13]. To our knowledge, no study has yet evaluated a combination of complete clinical characteristics, functional parameters and detailed HRCT assessment to predict baseline severity and long-term outcome of PFTs in SSc-ILD using LMM.

Our study was designed to address these issues. The aim of our study was to describe using LMM the evolution of FVC and DLCO in patients with SSc-ILD and to assess the baseline clinical, biological and HRCT predictors of this evolution.

## Patients and methods

### Patients

For inclusion, patients had to fulfil the following criteria: 1. American College of Rheumatology (ACR) criteria for SSc [14] and/or the LeRoy’s classification system [15]; 2. Presence of ILD on initial HRCT (independently confirmed by two experienced radiologists: JBF and ALH); 3. One available PFT performed within 2 months of the initial HRCT; 4. At least one additional PFT during follow up. The flowchart is depicted in S1 Fig. Our final study population included 75 consecutive patients fulfilling these criteria. This study was approved by the Commission Nationale de l’Informatique et de la Liberté (No. DC-2008-642) and was in accordance with current French legislation. Patients gave their consent and data were accessed anonymously.

### Clinical data

Baseline (i.e. the first visit to our institution) characteristics were recorded in the medical file at the time of the HRCT used to diagnose ILD and included the following: subtype of systemic sclerosis according to Leroy and Medsger [15], presence or history of digital ulcers (DU), arthralgia, synovitis, gastro-oesophageal reflux disease (GERD), New York Heart Association (NYHA) functional class and modified Rodnan skin score (mRSS). Disease durations were measured both from onset of Raynaud’s phenomenon (RP) and onset of first non-RP symptom and time of HRCT.

### PFTs

All patients underwent baseline PFT within 2 months of the baseline HRCT. All the serial PFTs were recorded.

Forced expiratory volume in 1 s (FEV1), and FVC were obtained by spirometry. Total lung capacity (TLC) was obtained by plethysmography or helium dilution test. DLCO was measured by single-breath nitrogen test. The percentage of predicted value for DLCO (DLCO%) was corrected for measured haemoglobin according to ATS/ERS guidelines [16, 17). The reference values used were those of the Official Statement of the European Respiratory Society [16, 17].

### Echocardiography

Forty-two patients had an available echocardiogram within 2 months of the first HRCT. During follow-up, we collected patients with right heart catheterization-proven PH and patients with a tricuspid regurgitation > 2.8 ms^-1^. Data on follow-up echocardiogram and right heart catheterization were available in 72/75 patients.

### HRCT

HRCT analysis is detailed in S1 Appendix. Briefly, HRCTs were independently reviewed by two radiologists experienced in interstitial lung diseases (JBF, ALH), blindly from clinical data. The staging described by Goh *et al*. (11) was established for each patient: ILD was classified as limited or extensive, based on ILD initial extent on HRCT with in intermediary cases the use of FVC. HRCT were scored at five levels, and the extent of ILD was classified as limited (under 20% of pulmonary parenchyma), or extensive (above 20%). For indeterminate cases, ILD was considered as extensive if FVC was below 70%, and limited if FVC was above 70%.

### Statistical analysis

All results of qualitative variables were expressed as the frequencies and percentages. Continuous variables were expressed as means and standard deviations in case of normal distribution and as medians and interquartile ranges otherwise. The normality of the distribution was tested using the Shapiro-Wilk test.

The evolution of FVC and DLCO during the study period was analyzed using linear mixed model with random coefficients [18]. This model is a generalization of the ANOVA for repeated measures. It is adapted in situations when the number and the time of repeated measurements differ between the subjects and it allows handling the correlations between the repeated measurements. In addition, the random intercept and the random slope allow taking into account the variability in the trajectories between the subjects and the fixed part of the model corresponds to the mean evolution. To look for an association between each baseline factors and the baseline value of FVC and DLCO, bivariate analyses were performed using linear regressions. Each parameter with a p-value < 0.20 was introduced in a multivariable linear model with forward selection (at the 0.05 significant level). Because FEV1 and FVC were strongly correlated, FEV1 was not introduced in multivariable model. The results of multivariable models were expressed as coefficients and their standard errors.

The association between each baseline factor and variation of DLCO was studied using a mixed model with random coefficients (bivariate analyses). A multivariable mixed model with forward selection (at 0.05 significant level) was performed on the variables having a significant level less than 0.2 in bivariate analyses. The time until the occurrence of a decline of FVC of more than 10% from baseline was analyzed using the Kaplan Meier method. The same method was used to analyze a decline of DLCO of more than 15% from baseline.

Statistical analysis was performed by SAS software (SAS Institute version 9.3).

## Results

Baseline demographic data, extent on HRCT and characteristics of PFT are reported in Table 1. Mean age was 52.0±15.8 (range: 18–82). ILD was limited in 76% and extensive in 24% of patients. An echocardiogram was available in 42 patients (56%) at the diagnosis of ILD. Two patients had right heart catheterization-proven precapillary PH at the diagnosis of SSc-ILD. Twenty three patients were treated (2 before 3 months and 21 after 3 months of follow up). Among these 23 patients, 19 received cyclophosphamide, two mycophenolate mofetil, one methotrexate and one autologous stem cell transplantation.

**Table 1:** Demographic data and ILD characteristics at baseline.

Variable	Value
Age, years	52.0±15.8
Sex, n M:F	18:57
BMI, kg/cm^2^	24.6±4.1
Disease duration since first Raynaud’s phenomenon, years	6.7±8.5
Disease duration since first non-Raynaud’s phenomenon, years	2.8±3.8
Ethnicity	
Caucasian, n(%)	67 (89)
Non-Caucasian, n(%)	8 (11)
Active tobacco use, n(%)	6 (8)
Type of scleroderma	
Limited, n(%)	52 (69)
Diffuse, n(%)	23 (31)
mRSS	11.9±10.9
Anticentromere antibodies/antitopoisomerase antibodies/anti RNP antibodies, n(%)	9 (12%)/41 (55%)/2 (3%)
NYHA functional class I-II/III-IV, n(%)	60 (80%)/15 (20%)
Respiratory symptoms leading to ILD diagnosis, n(%)	16 (24%)
GERD, n(%)	57 (76%)
Digital ulcers (presence or history), n(%)	25 (33%)
Arthralgia/synovitis, n(%)	27 (36%)/11 (15%)
PFT	
FVC (% predictive value)	90.0±19.9
DLCO (% predictive value)	67.2±23.9
FEV1 (% predictive value)	88.8±19.8
FEV1/FVC, %	78.8±12.5
CRP, mg/L	7.5±11.6
Hb, g/dL	13.0±1.4
Creatinine, mg/L	77.9±21.4
HRCT	
ILD extent (% of parenchyma)	20.9±18.8
Reticular pattern extent (% of parenchyma)	6.0±9.5
Proportion of ground-glass opacification (% of ILD)	74.4±27.7
Coarseness	3.2±2.7
Global score of bronchectasia	2.5±2.8
Emphysema extent (% of parenchyma)	0.26±0.84
ILD extension according to Goh *et al*.: limited/extensive, n(%)	57 (76%) / 18 (24%)
ILD grade 1/2/3, n(%)	47 (63%)/21 (28%)/7 (9%)

Results are expressed as mean±standard deviation, number (percentage). M: males; F: females; BMI: body mass index; mRSS: modified Rodnan skin score; ILD: interstitial lung disease; GERD: gastro-oesophageal reflux disease; PFT: pulmonary function tests; FVC: forced vital capacity; DLCO: diffusion capacity for carbon monoxide; FEV1: forced expiratory volume in 1 second; CRP: C-reactive protein; Hb: haemoglobin, HRCT: high resolution computed tomography.

### Evolution

**Fig 1:**
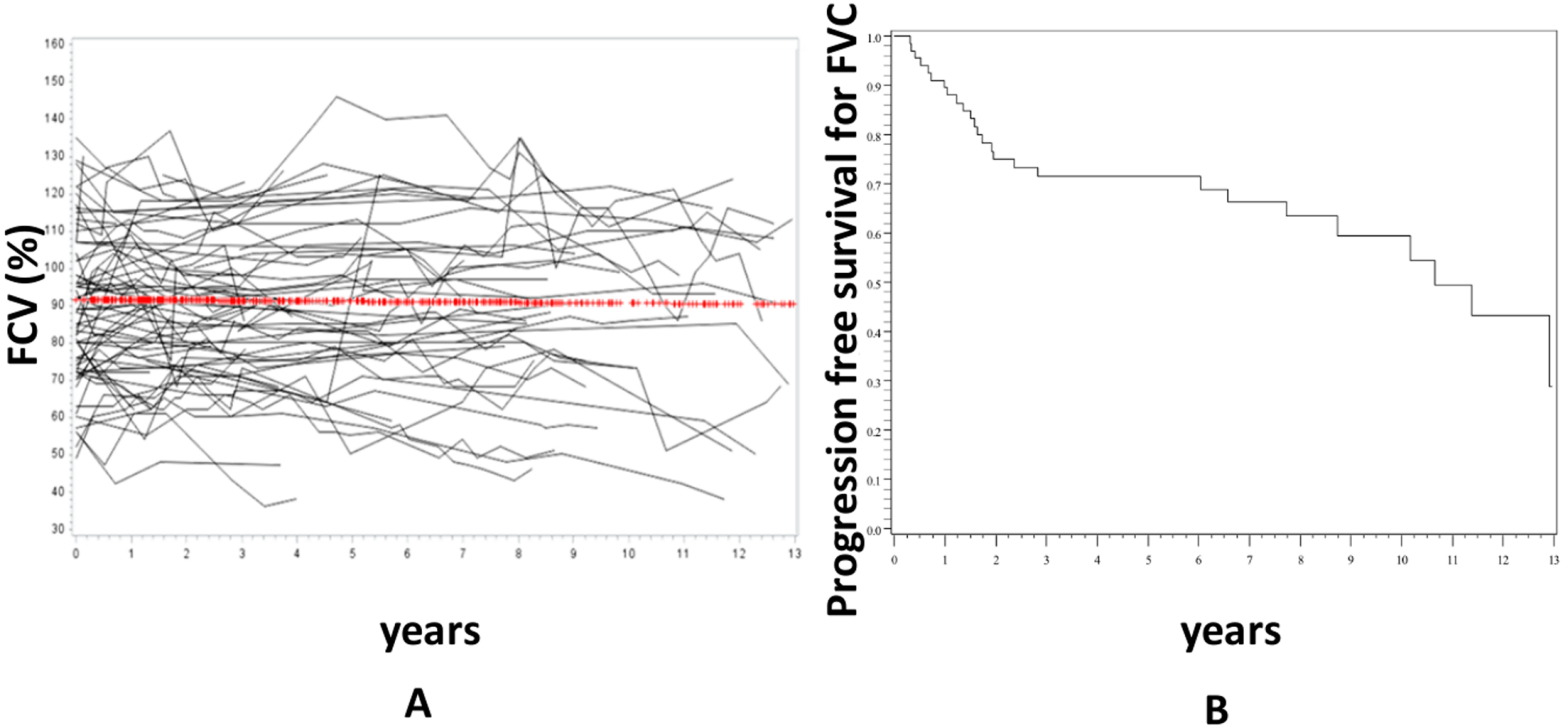
Evolution of FVC during the follow up. Panel A represents the individual data of the 75 patients and the red line is the trajectory of FVC using the linear mixed model with random effect. There was no significant change of FVC over time. Panel B is the progression-free survival of FVC. Progression was defined as a decline of ≥10% of baseline FVC.

Mean follow-up was 6.4±4.2 years. Patients underwent a median number of 5 (range 1–19) PFTs/patient during the follow up and 557 PFTs were analysed overall. Median delay between 2 PFTs was 7.2 months. There was no significant variation in FVC over the study period (-0.1±0.3%/year, p = 0.71) (Fig 1), whereas 29% of patients had experienced a significant decline of FVC of more than 10% from baseline after 6 years of follow up (Fig 1).

**Fig 2:**
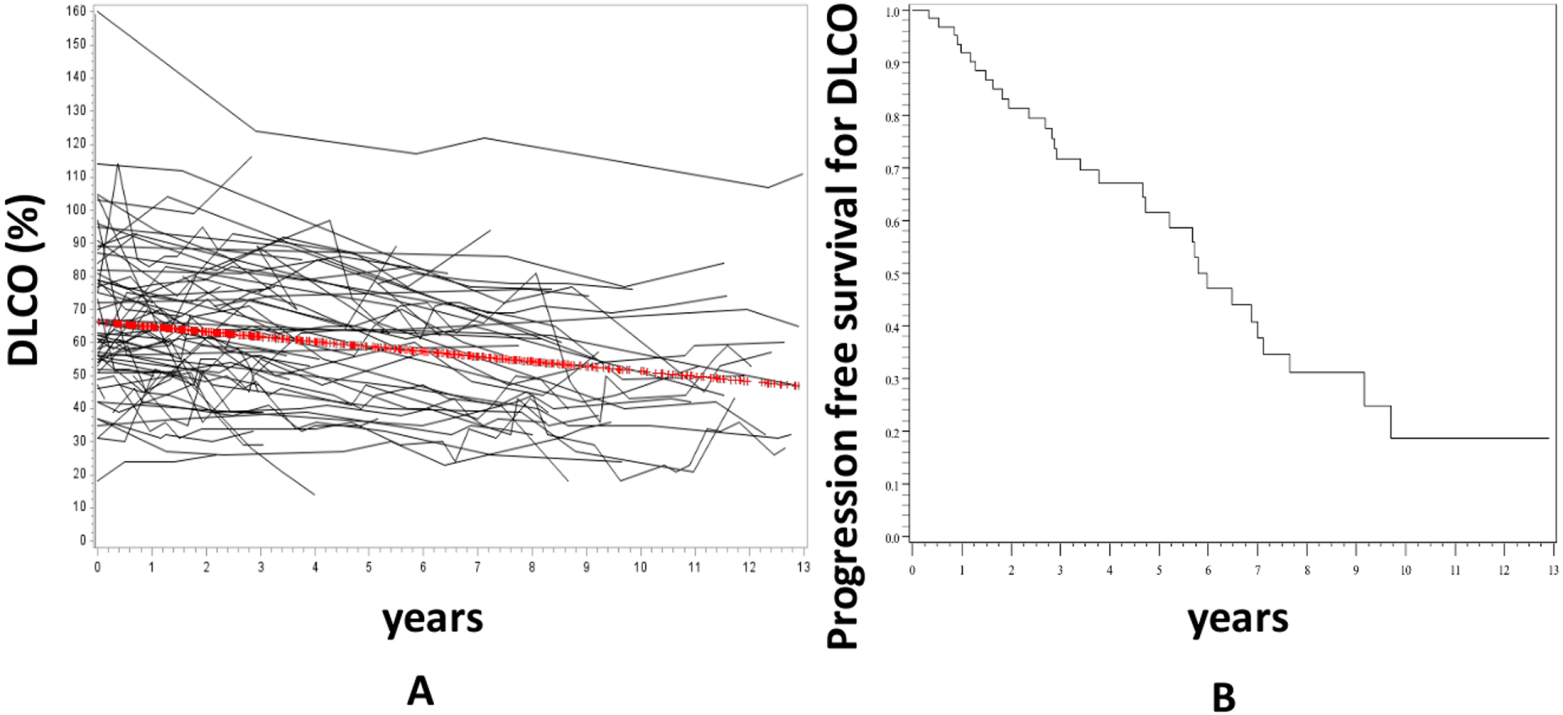
Evolution of DLCO during the follow up. Panel A represents the individual data of the 75 patients and the red line is the trajectory of DLCO using the linear mixed model with random effect. DLCO significantly decreased over time (-1.5±0.3%/year (p<0.0001). Panel B is the progression-free survival of DLCO. Progression was defined as a decline of ≥15% of baseline DLCO.

Conversely, DLCO declined significantly by 1.5±0.3%/year (p<0.0001) (Fig 2). After 6 years of follow up, 53% of patients had experienced a significant decline of DLCO of more than 15% from baseline (Fig 2).

Eleven patients died during the follow up, and the survival rate at 10 years was 85%. Twenty-three patients needed an immunosuppressive drug due to the evolution of ILD, after a median time of 11 months (range 3–132). Fourteen patients developed a right heart catheterization-proven precapillary PH during the follow up.

As the FVC did not change significantly during follow up, we assessed the baseline predictors of the initial FVC but not of the slope of FVC overtime (instead, we analyzed the predictors of confirmed decline of FVC of more than 10% from baseline) while we assessed the baseline predictors of both the initial DLCO and the slope of DLCO over time.

### Baseline predictors of initial FVC

NYHA functional class III or IV, baseline DLCO lower than 70% and FEV1 lower than 75% were associated with a lower FVC at baseline in bivariate analysis (S1 Table)). Concerning HRCT characteristics, an extensive ILD and ILD lesions involving more than 30% of lung parenchyma were associated with a lower baseline FVC. There was a trend for extension of reticulation ≥5% and global score of bronchectasia ≥1.5 to be associated with a lower baseline FVC. In multivariate analysis, baseline DLCO and ILD extension according to Goh’s staging system were independent predictors of baseline FVC (S2 Table).

### Predictors of decline in FVC

As the overall FVC curve was flat, we did not assess the baseline predictors of the FVC slope. Instead, we studied the predictors of a significant decline of more than 10% of FVC from baseline). Results are shown in S3 Table. The only predictors were baseline DLCO and ILD extension as well as the use of immunosuppressants during follow up.

### DLCO

#### Baseline predictors of initial DLCO

On bivariate analysis, presence of respiratory symptoms leading to HRCT, FVC lower than 80% and FEV1 lower than 75% and ummunosuppressants use during follow up were associated with a lower DLCO at baseline (Table 2). Concerning HRCT characteristics, an extensive ILD and ILD lesions involving more than 30% of lung parenchyma were associated with a lower DLCO at baseline.

**Table 2:** Bivariate analysis of parameters associated with baseline value and slope of DLCO.

Variable		Mean baseline DLCO expressed as % of predicted value	Standard error	p	Slope of DLCO (mean/yr)	Standard error	p
Age at diagnosis (years)	<50	69.8	4.3	0.42	-1.32	0.39	0.51
	>50	64.9	4.1		-1.70	0.41	
Sex	F	65.8	3.4	0.40	-1.72	0.31	0.17
	M	71.6	6.0		-0.83	0.56	
Disease duration since first Raynaud’s phenomenon	<4 years	63.4	4.3	0.29	-1.08	0.40	0.16
	> 4 years	69.7	4.1		-1.85	0.38	
Disease duration since first non-Raynaud’s phenomenon	<1.5 years	70.3	4.5	0.38	-1.14	0.38	0.13
	> 1.5 years	64.8	4.2		-1.98	0.39	
Ethnicity	Caucasian	66.4	3.2	0.55	-1.50	1.10	0.96
	Non-Caucasian	72.3	9.1		-1.46	0.30	
Type of SSc	Limited	68.9	3.6	0.40	-1.50	0.34	0.93
	Diffuse	63.5	5.4		-1.45	0.52	
mRSS	<6	66.7	4.6	0.99	-1.43	0.46	0.79
	>6	66.6	4.5		-1.60	0.41	
Anticentromere antibodies	No	68.2	3.2	0.38	-1.50	0.30	0.99
	Yes	60.3	8.5		-1.49	0.98	
Anti-topoisomerase I	No	64.8	4.5	0.47	-1.06	0.47	0.27
	Yes	69.1	3.9		-1.71	0.36	
Dyspnoea (NYHA)	I or II	69.3	3.3	0.15	-1.38	0.30	0.29
	III or IV	58.3	6.8		-2.17	0.69	
Respiratory symptoms leading to ILD diagnosis	No	70.7	3.4	0.006*	-1.58	0.34	0.54
	Yes	51.1	6.0		-1.13	0.64	
GERD	No	64.1	6.0	0.54	-1.47	0.64	0.97
	Yes	68.3	3.4		-1.50	0.32	
Digital ulcers (presence or history)	No	64.0	3.6	0.12	-1.01	0.33	0.01*
	Yes	76.3	5.0		-2.45	0.45	
Arthralgia	No	67.8	3.8	0.80	-1.64	0.35	0.50
	Yes	66.3	4.9		-1.25	0.46	
Synovitis	No	67.0	3.2	0.83	-1.63	0.31	0.23
	Yes	68.9	8.0		-1.04	0.65	
Baseline FVC (%)	<80	56.2	5.4	0.014*	-1.28	0.53	0.58
	>80	72.2	3.4		-1.64	0.36	
Baseline FVC (%)	<70	52.1	8.8	0.06	-1.29	0.46	0.67
	>70	69.6	3.1		-1.48	0.30	
Baseline DLCO (%)	<70	51.7	2.4	<0.0001	-0.91	0.24	0.02
	>70	89.1	2.90		-1.68	0.32	
Baseline FEV1 (%)	<75	52.7	5.6	0.004*	-1.28	0.52	0.73
	>75	72.2	3.2		-1.49	0.34	
Baseline FEV1/FVC (%)	<75	69.4	6.5	0.62	-0.52	0.77	0.22
	>75	65.6	3.6		-1.54	0.32	
CRP (mg/L)	<10	66.3	3.9	0.99	-1.51	0.31	0.17
	>10	66.3	7.2		-2.48	0.63	
Hb (g/dL)	<13	66.1	4.7	0.87	-1.72	0.44	0.38
	>13	67.2	4.6		-1.17	0.46	
Creatinine (μmol/l)	<70	67.8	5.6	0.77	-0.84	0.56	0.15
	>70	65.8	3.8		-1.79	0.35	
Extension of ILD (%)	<30	72.8	3.1	0.001*	-1.68	0.34	0.23
	>30	50.3	5.5		-0.93	0.53	
Extension of ILD (%)	<20	74.1	3.48	0.002*	-1.62	0.38	0.45
	>20	55.6	4.54		-1.19	0.43	
Extension of reticulations (%)	<5	68.2	3.6	0.64	-1.14	0.36	0.18
	>5	65.2	5.2		-1.89	0.42	
Proportion of ground-glass opacification (%)	<70	62.8	4.8	0.24	-1.51	0.43	0.93
	>70	70.0	3.8		-1.46	0.38	
Coarseness	<3.6	69.8	4.2	0.39	-1.30	0.41	0.54
	>3.6	64.6	4.2		-1.65	0.39	
Global score of bronchectasia	<1.5	70.7	4.3	0.27	-1.42	0.49	0.90
	>1.5	64.1	4.1		-1.50	0.35	
Emphysema extent (%)	0	67.9	3.2	0.55	-1.52	0.31	0.75
	>0	62.5	8.5		-1.26	0.79	
ILD extension according to Goh *et al*.	Limited	71.9	3.2	0.004*	-1.69	0.33	0.21
	Extensive	51.8	5.8		-0.90	0.53	
ILD grade	1	69.6	3.6	0.27	-1.05	0.45	0.20
	2 or 3	62.7	5.1		-1.79	0.36	
PH by right heart catheterization at baseline or during follow up	0	69.3	3.3	0.15	-1.19	0.31	0.06
	1	58.1	6.9		-2.37	0.55	
Tricuspid regurgitation>2.8 ms^-1^ at baseline or during follow up	0	70.0	3.6	0.15	-1.10	0.34	0.06
	1	60.5	5.5		-2.17	0.45	
Immunosuppressants use during follow up	No	72.7	2.95	0.003	-1.32	0.35	0.64
	Yes	53.4	4.42		-1.61	0.49	

FVC: forced vital capacity; SSc: systemic sclerosis; ILD: interstitial lung disease; GERD: gastro-oesophageal reflux disease; mRSS: modified Rodnan skin score; DLCO: diffusion capacity for carbon monoxide; FEV1: forced expiratory volume in 1 second, CRP: C-reactive protein; Hb: haemoglobin; PH: precapillary pulmonary hypertension

*p-value less than 0.05

#### Baseline predictors of DLCO evolution

The presence or history of DU at SSc-ILD diagnosis as well as a baseline DLCO>70% were associated with a faster decline in DLCO over the study period (Table 2). There was a trend for the presence at baseline or during follow up of right-heart catheterization precapillary PH as well as tricuspid regurgitation ≥ 2.8 ms^-1^ to be associated with a faster decline in DLCO overtime in bivariate analysis (p = 0.06). Conversely, neither age, gender, disease duration, subtypes of SSc or antibodies, presence of GERD nor extension of ILD and reticulations were associated with a different evolution of DLCO.

#### Predictors of baseline DLCO and DLCO evolution: multivariate analysis

**Fig 3:**
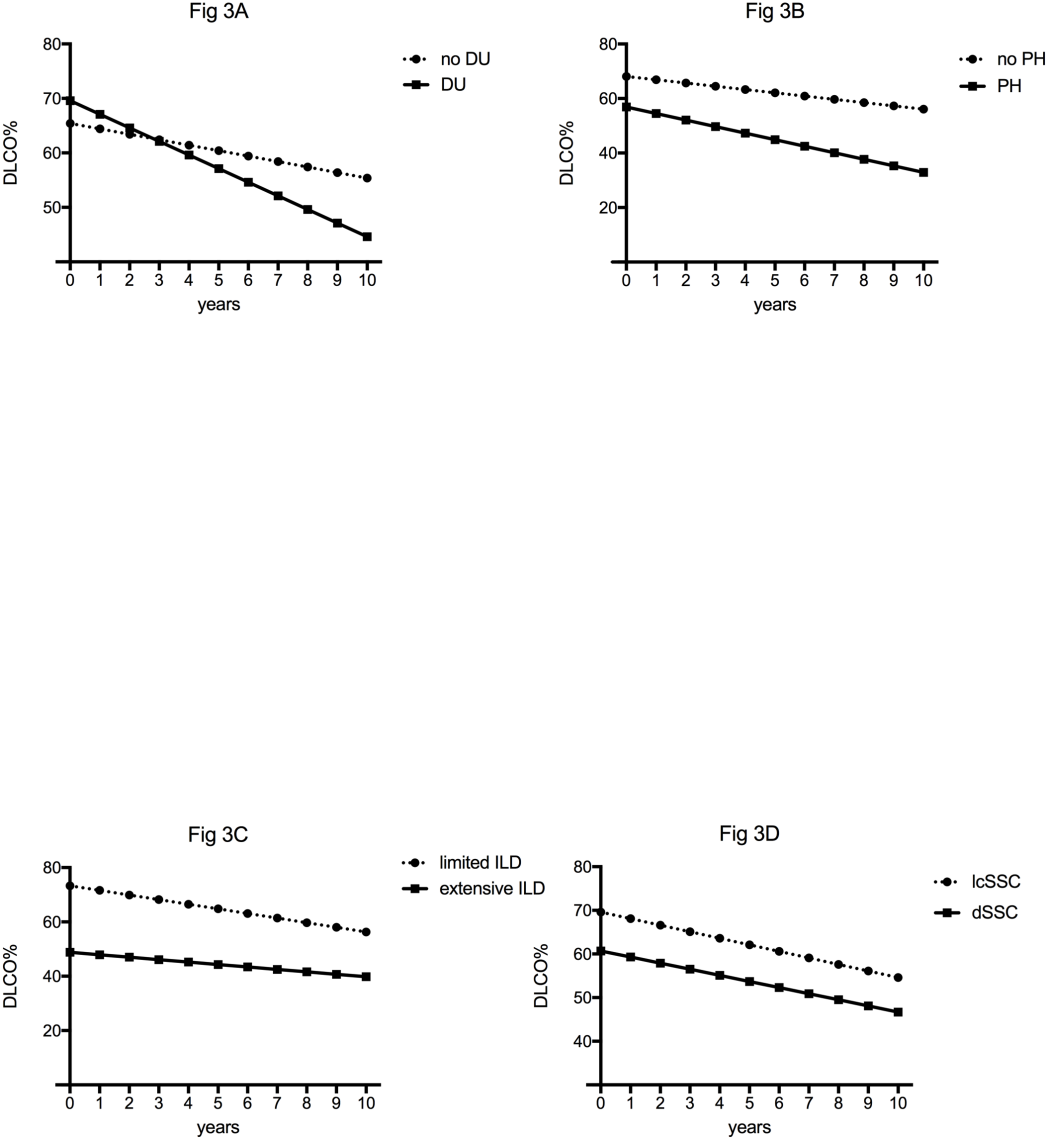
Trajectories of DLCO of patients with various clinical characteristics, using the linear mixed model with random effect. Panel A. Patients with presence or history of digital ulcers (DU) had a significantly higher baseline DLCO% but a faster decline of DLCO than patients without presence or history of digital ulcers (no DU) in multivariate analysis: -2.45±0.45 vs -1.01±0.33%/year (p = 0.01). Panel B. Patients with a right heart catheterization-proven precapillary pulmonary hypertension at baseline or during follow-up (PH) had a similar baseline DLCO% but a faster decline of DLCO than patients without a right heart catheterization-proven precapillary pulmonary hypertension at baseline or during follow-up (no PH) in multivariate analysis: -2.37±0.55 vs -1.19±0.31%/year (p = 0.049). Panel C. Patients with a limited ILD according to Goh *et al*. had a significantly higher baseline DLCO than patients with an extensive ILD but the slope of decrease in DLCO was similar in both groups (-1.69±0.33 vs -0.90±0.53%/year (p = 0.21). Panel D. Patients with a limited cutaneous SSc (lcSSc) had similar baseline DLCO and slope of decrease in DLCO than patients with diffuse SSc (dSSc) (p = 0.40 and p = 0.93, respectively).

On multivariate analysis, presence of respiratory symptoms, absence of DU and lower baseline FVC were associated with a lower baseline DLCO (S4 Table). On multivariate analysis, a faster decline in DLCO was associated with the presence or history of DU at diagnosis as well as PH by right heart catheterization at baseline or during follow up (S5 Table and Fig 3).

## Discussion

Our study describes the evolution of FVC and DLCO as well as the predictors of baseline PFTs severity and outcome in an unselected population of 75 consecutive patients with SSc-ILD. The first result is that there was no significant change of FVC over time in our population. This result was quite unexpected as some studies found that FVC significantly declined during the follow up in SSc patients with ILD [11, 19]. There are some possible explanations for these discrepancies. The most probable one is that Goh *et al*. performed a time to decline analysis rather than directly measuring the decline rate of FVC [11]. We also found in our study that a proportion of our patients (29%) experienced a drop of more than 10% of FVC during the first 72 months of follow up (Fig 2) but the overall FVC curve was flat when modelled by LMM. This suggests that defining a worsening of ILD by a drop of FVC can be somehow misleading as some patients spontaneously improve over time. In another study, using a different statistical approach, Moore et al. also found that the annual rate of decline in FVC was low (0.08±3 L/year) [20]. One could argue that this overall stable FVC observed in our population could be a misleading mean of various and opposite trajectories of homogenous groups, for example of one group with worsening FVC and another with improving FVC. To address these issues, we perform a Latent Class Linear Mixed Model (LCLMM) analysis. The LCLMM consists to assume that the population is divided in a finite number of groups called the latent classes. In each latent class, the trajectories of the patients are close to each other while the trajectories of patients in different classes tend to be dissimilar. This procedure allows to detect homogenous groups of trajectories without any a priori assumption [21]. This analysis showed that no homogenous groups of trajectories could be identified among our patients. This suggest that the flat curve of FVC is not due to a misleading mean of various and opposite trajectories of homogenous groups but to a highly intraindividual variation of FVC leading to an overall flat curve. This result is in line with Bouros et al. study which showed that there was little overall decline in FVC overtime although large changes were observed in individual patients [22]. Altogether, these results could suggest that FVC, which is used as a surrogate marker and primary endpoint in many clinical trials in SSc-ILD, could be both highly variable in a same patient [23] and overall stable overtime in an unselected population. It should be highlighted that a recent study by Goh et al. suggested that a short term variation in FVC was associated with mortality in patients with an extensive ILD [24]. Whether FVC is the best outcome in clinical trials, especially in patients with extensive ILD if there is a cohort enrichement, remain to be firmly established.

A few parameters were associated with a lower FVC and a lower DLCO at baseline. Some were fully expected, such as the association with respiratory symptoms or significant dyspnea. As anticipated, an extensive ILD was associated with a lower FVC and DLCO at baseline. These results are in line with Goh *et al*.’s study, which demonstrated a negative correlation between ILD extent on HRCT and baseline FVC% (r = -0.40, p<0.0005) [11].

Concerning FVC outcome, we were not able to assess the factors associated with a faster decline of FVC slope as the curve was overall flat. When assessing the factors associated with a higher risk if a significant deline of FVC of more than 10% from baseline, we found that the initial extension of ILD on HRCT scan as well as baseline DLCO was significantly associated with this risk. These results have already been reported in Goh’s study [11].

Concerning DLCO outcome, the main result is that there was a significant decline over time. Some of the factors associated with a faster decline of DLCO need to be discussed. DU are the hallmark of vasculopathy in SSc [25–27]. Interestingly, a recent study found that presence of DU and density loss in capillaries on capillaroscopy were associated with a greater decrease in DLCO than in FVC in SSc [28]. We could therefore suggest that patients with DU have a more severe vasculopathy and therefore a faster decrease of DLCO over time, as shown in our study. Unfortunately, we did not have the capillaroscopy data and were therefore unable to assess whether they could be associated with baseline DLCO as well as DLCO slope. DLCO decrease could also reflect the pulmonary vascular involvement of SSc. Indeed, we found that having or developing a right heart catheterization-proven precapillary PH was associated with a faster decline in DLCO. Decline in DLCO is known to precede the onset of PH in SSc [29, 30]. Altogether, our results suggest that a faster decline in DLCO in patients with SSc-ILD is associated with more severe vascular events.

Interestingly neither gender nor subtypes of SSc was associated with a different baseline FVC and DLCO or with a different DLCO outcome. Diffuse SSc is consistently associated with a higher risk of ILD. However, once ILD is present, there are no convincing data to suggest that patients with diffuse SSc have a more severe and aggressive ILD than patients with limited SSc [31].

In our study, the initial extension of ILD on HRCT according to Goh’s staging system was associated with the baseline FVC and DLCO but not with the DLCO slope overtime. This result was unexpected as several studies have showed that an extensive ILD was associated with a lower progression-free survival in SSc-ILD [11, 12, 20]. However, in these studies, progression was defined as decrease of more than 10% of FVC and/or more than 15% of DLCO during serial PFTs [11]. This definition could be slightly misleading as the variation of FVC during the follow up can sometimes meet the definition of a progression by a drop of more than 10% while the FVC curve is overall flat. The main explanation is the intraindividual variation of FVC and DLCO in patients with or without any treatment which has been shown previously [22] and is confirmed here.

Our study was not designed to study the role of immunosuppressants in the evolution of ILD. However, we assessed wether the use of immunosuppressant during follow-up was associated with any significant change in DLCO. We did not find an association between immunosuppressant use and the slope of DLCO during follow up. As the overall FVC curve was flat, we assessed the immunosuppressants use during follow up as a predictor of confirmed decline in FVC and found that it was a significant predictor. We interpret these data with caution as the study was not designed to assess the role of immunosuppressants in SSc-ILD. The higher risk of decline of FVC with the immunosuppressants use during follow up is probably explained because we usually start these treatments in worsening ILD.

Our study has several limitations. Firstly, the retrospective design of the study was responsible for some missing data. Secondly, mean baseline FVC was high and might not be representative of more severely affected SSc-ILD patients. Secondly, the quite unexpected stability of FVC over the study period did not allow us to search for prognostic factors for FVC evolution. Third, the sample size of our population was rather small and the mean baseline FVC high. Therefore, our population could not be the representative of more severe SSc-ILD and no firm conclusion can be drawn on the usefulness of categorical lung function changes. Larger studies including more patients with severe SSc-ILD patients are thus needed.

## Conclusions

In conclusion, this study confirms that LMM is an effective way of modelling the evolution of PFTs in patients with SSc-ILD, and of evaluating predictors for this evolution. In our population, FVC remained stable over the study period whereas DLCO declined significantly. This highlights the difficulty in finding robust predictors for FVC evolution in SSc-ILD and could explain why randomized control trials using FVC as the primary endpoint either failed or showed modest benefit. This also suggests that systematic HRCTs mostly diagnose patients with stable FVC, which is an important finding in daily practice. An extensive SSc-ILD on HRCT was associated with a lower baseline FVC and DLCO but not with evolution over time. For DLCO, presence of DU and having or developing a right heart catheterization-proven precapillary PH were associated a faster decline. This suggests that a decline in DLCO is associated with more severe vascular events in SSc-ILD. Noteworthy, neither gender nor SSc subtype was a predictor of initial severity or outcome.

## Supporting information

S1 Fig Flowchart of the study. (TIF)

S1 Appendix HRCT analysis. (DOCX)

S1 Table Bivariate analysis of parameters associated with baseline value of FVC. (DOCX)

S2 Table Multivariate analysis of parameters associated with baseline value of FVC. (DOCX)

S3 Table Decline in FVC, expressed as hazards ratio with 95% confidence intervals, in relation to baseline data (univariate analysis). (DOCX)

S4 Table Multivariate analysis of parameters associated with baseline value of DLCO. (DOCX)

S5 Table Multivariate analysis of parameters associated with slope of DLCO. (DOCX)
