# Supplementary figures and images for: Predictors of lung function test severity and outcome in systemic sclerosis-associated interstitial lung disease
Source: PLoS One. 2017 Aug 1;12(8):e0181692. doi: 10.1371/journal.pone.0181692 (PMC5538660; doi:10.1371/journal.pone.0181692)

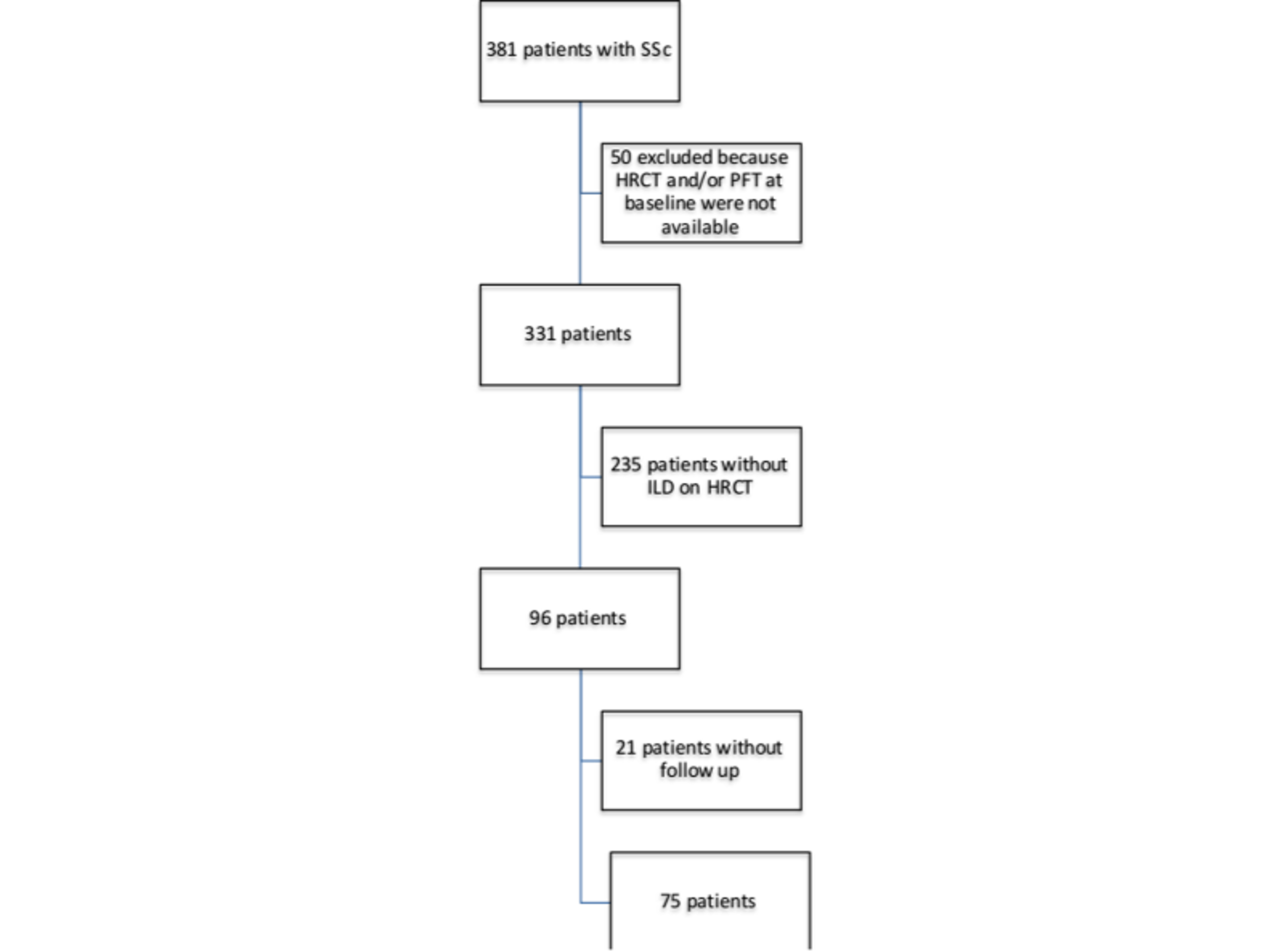

Supplement: S1 Fig — (TIF) [file pone.0181692.s001.tif]
