# Supplementary material for: Predictors of lung function test severity and outcome in systemic sclerosis-associated interstitial lung disease
Source: PLoS One. 2017 Aug 1;12(8):e0181692. doi: 10.1371/journal.pone.0181692 (PMC5538660; doi:10.1371/journal.pone.0181692)
Supplement: S1 Appendix — (DOCX) [file pone.0181692.s002.docx]

# Supplemental Appendix 1

## HRCT

HRCT scans were independently reviewed by two radiologists experienced in interstitial lung diseases (JBL, ALH), blindly from clinical data. HRCT scans were reviewed at the following six levels, adapted from ^1^: 1, origin of great vessels; 2, carina; 3, pulmonary venous confluence; 4, between levels 3 and 5; 5, 1 cm above the right hemidiaphragm; and 6, 2 cm below the right hemidiaphragm. When compared to the original study ^1^ which focused on the first 5 levels, we decided to add a sixth one because SSc-ILD predominates in the lower lobes, which, in our opinion, was not sufficiently captured by the first 5 levels.

At each level, the following features were quantified:

(A) the overall extent of ILD, including both ground-glass opacification and reticular opacification, estimated to the nearest 5%; (B) the relative proportions (up to a total of 100%) of reticular pattern and ground-glass opacification in the overall interstitial lung disease; (C) the coarseness of fibrosis, quantified as follows: 0 – ground-glass opacification alone, 1 – fine intralobular fibrosis, 2 – microcystic reticular pattern comprising air spaces smaller than or equal to 4 mm in diameter, 3 – a macrocystic reticular pattern comprising air spaces larger than4 mm in diameter; (D) the extent of emphysema, which was defined as areas of decreased attenuation usually without discrete walls of non-uniform distribution causing permeative destruction of lung parenchyma, quantified to the nearest 5%. Then, all HRCT scans were reviewed to define a semi-quantitative evaluation of ILD on the whole scan as follows: grade 1, predominant ground-glass opacification; grade 2, equal proportions of ground-glass opacification and reticular pattern; grade 3, predominant reticular pattern ^2^. Ground-glass opacification was defined as a hazy increase in lung parenchymal attenuation, with preservation of bronchial and vascular markings. A reticular pattern was defined as innumerable interlacing line shadows that were fine, intermediate or coarse, with associated distortion of the lung architecture.

Discrepancies in the extent of ILD, the relative percentages of a reticular pattern, ground-glass opacification or emphysema of more than 20% at any level were reviewed jointly and resolved with consensus evaluation. Similarly, a difference of more than one grade at each level for the coarseness score and the overall grade of fibrosis was resolved by means of joint review. For each patient, the extension of ILD and of emphysema was derived by averaging the scores at each level, as assessed by the two observers; the mean value was used in the analysis. The proportion of ground-glass opacification was computed by dividing the overall extent of ground-glass opacification by the extent of ILD. Overall extent of ground-glass opacification was obtained by averaging the extent of ground-glass opacification at each level.

The overall coarseness score for each patient was derived by summing the scores at the six levels (minimum score, 0; maximum score, 18). To prevent spurious reduction in the coarseness score, it was adjusted proportionately to a six-level score in patients with no disease in one or more HRCT sections. Thus, in a patient with no disease in one section, a coarseness of 8 would be adjusted by 6/5 and would result in an overall coarseness score of 9.6.

For each HRCT level, traction bronchiectasis was scored as follows: 0 – none, 1 – mild, 2 – moderate, 3 – severe A summed traction bronchiectasis score was computed for each patient by summing the score of the six levels. A global score was obtained by adjusting the summed traction bronchiectasis score in patients with no ILD in one or more HRCT sections, as was done for the overall coarseness score ^3^.

Finally, the staging described by Goh *et al.* was established for each patient: ILD was classified as limited or extensive, based on ILD initial extent on HRCT with in intermediary cases the use of FVC. HRCT were scored at five levels, and the extent of ILD was classified as limited (under 20% of pulmonary parenchyma), or extensive (above 20%). For indeterminate cases, ILD was considered as extensive if FVC was under 70%, and limited if FVC was above 70%.

Each HRCT was reviewed to define a semi-quantitative evaluation of ILD on the whole scan as follows: grade 1, predominant ground glass opacities; grade 2, equal proportions of ground-glass opacities and reticular pattern; grade 3, predominant reticular pattern ^2^.

REFERENCES

1. Desai SR, Veeraraghavan S, Hansell DM, et al. CT features of lung disease in patients with systemic sclerosis: comparison with idiopathic pulmonary fibrosis and nonspecific interstitial pneumonia. *Radiology.* 2004;232(2):560-567.

2. Collins CD, Wells AU, Hansell DM, et al. Observer variation in pattern type and extent of disease in fibrosing alveolitis on thin section computed tomography and chest radiography. *Clin Radiol.* 1994;49(4):236-240.

3. Edey AJ, Devaraj AA, Barker RP, Nicholson AG, Wells AU, Hansell DM. Fibrotic idiopathic interstitial pneumonias: HRCT findings that predict mortality. *Eur Radiol.* 2011;21(8):1586-1593.
