# Supplementary material for: Predictors of lung function test severity and outcome in systemic sclerosis-associated interstitial lung disease
Source: PLoS One. 2017 Aug 1;12(8):e0181692. doi: 10.1371/journal.pone.0181692 (PMC5538660; doi:10.1371/journal.pone.0181692)
Supplement: S1 Table — (DOCX) [file pone.0181692.s003.docx]

S1 Table. Bivariate analysis of parameters associated with baseline value of FVC

| **Variable** | | **Mean baseline FVC expressed as % of predicted value** | **Standard error** | **p** |
| --- | --- | --- | --- | --- |
| Age at diagnosis (years) | <50 | 87.1 | 3.4 | 0.24 |
|  | >50 | 92.7 | 3.3 |  |
| Sex | F | 89.2 | 2.7 | 0.56 |
|  | M | 92.6 | 5.0 |  |
| Disease duration since first Raynaud’s phenomenon | <4 years | 89.6 | 3.7 | 0.60 |
|  | ≥4 years | 92.3 | 3.5 |  |
| Disease duration since first non-Raynaud’s phenomenon | <1.5 years | 91.6 | 3.8 | 0.91 |
|  | ≥1.5 years | 91.0 | 3.5 |  |
| Ethnicity | Caucasian | 91.4 | 7.0 | 0.11 |
|  | Non-Caucasian | 79.3 | 2.5 |  |
| Type of SSc | Limited | 90.2 | 2.9 | 0.87 |
|  | Diffuse | 89.4 | 4.4 |  |
| mRSS | <6 | 91.8 | 3.5 | 0.34 |
|  | ≥6 | 81.1 | 3.5 |  |
| Anticentromere antibodies | No | 88.6 | 2.5 | 0.11 |
|  | Yes | 100.6 | 7.0 |  |
| Anti-topoisomerase I | No | 90.5 | 3.6 | 0.83 |
|  | Yes | 89.5 | 3.3 |  |
| Dyspnoea (NYHA) | I or II | 92.6 | 2.6 | 0.027* |
|  | III or IV | 79.5 | 5.2 |  |
| Respiratory symptoms leading to ILD diagnosis | No | 94.0 | 2.9 | 0.06 |
|  | Yes | 83.1 | 5.0 |  |
| GERD | No | 94.1 | 4.8 | 0.34 |
|  | Yes | 88.7 | 2.7 |  |
| Digital ulcers | No | 89.3 | 2.9 | 0.69 |
|  | Yes | 91.3 | 4.2 |  |
| Arthralgia | No | 90.7 | 3.1 | 0.71 |
|  | Yes | 88.9 | 3.9 |  |
| Synovitis | No | 90.3 | 2.6 | 0.91 |
|  | Yes | 89.6 | 6.4 |  |
| Baseline DLCO (%) | <80 | 87.5 | 2.7 | 0.005* |
|  | ≥80 | 102.9 | 4.6 |  |
| Baseline DLCO (%) | <70 | 85.6 | 3.08 | 0.005 |
|  | ≥70 | 99.7 | 6.61 |  |
| Baseline FEV1(%) | <75 | 69.8 | 3.4 | <0.0001* |
|  | ≥75 | 98.7 | 2.2 |  |
| Baseline FEV1/FVC (%) | <75 | 94.1 | 5.5 | 0.53 |
|  | >75 | 90.2 | 2.9 |  |
| CRP (mg/L) | <10 | 89.7 | 3.1 | 0.63 |
|  | ≥10 | 86.6 | 5.6 |  |
| Hb (g/dL) | <13 | 86.5 | 3.6 | 0.39 |
|  | ≥13 | 91.0 | 3.6 |  |
| Creatinine (µmol/l) | <70 | 93.0 | 4.3 | 0.30 |
|  | ≥70 | 87.5 | 3.1 |  |
| Extension of ILD (%) | <30 | 93.3 | 2.7 | 0.016* |
|  | ≥30 | 80.3 | 4.5 |  |
| Extension of ILD (%) | <20 | 92.6 | 3.0 | 0.16 |
|  | ≥20 | 85.6 | 3.9 |  |
| Extension of reticulations  (%) | <5 | 93.3 | 2.9 | 0.052 |
|  | ≥5 | 83.6 | 4.0 |  |
| Proportion of ground-glass opacification (%) | <70 | 88.0 | 3.7 | 0.48 |
|  | ≥70 | 91.4 | 3.1 |  |
| Coarseness | <3.6 | 92.9 | 3.4 | 0.22 |
|  | ≥3.6 | 87.0 | 3.4 |  |
| Global score of bronchectasia | <1.5 | 94.8 | 3.5 | 0.063 |
|  | ≥1.5 | 85.9 | 3.2 |  |
| Emphysema extent (%) | 0 | 90.7 | 2.6 | 0.45 |
|  | >0 | 85.2 | 6.7 |  |
| ILD extension according to Goh *et al*. | Limited | 93.7 | 2.6 | 0.005* |
|  | Extensive | 78.5 | 4.6 |  |
| ILD grade | 1 | 92.0 | 3.0 | 0.27 |
|  | 2 or 3 | 86.5 | 3.9 |  |
| PH by right heart catheterization at baseline or during follow up | 0 | 90.9 | 2.8 | 0.85 |
|  | 1 | 89.8 | 5.4 |  |
| Tricuspid regurgitation≥2.8 ms^-1^ at baseline or during follow up | 0 | 91.1 | 3.0 | 0.82 |
|  | 1 | 89.9 | 4.4 |  |

FVC: forced vital capacity; SSc: systemic sclerosis; ILD: interstitial lung disease; GERD: gastro-oesophageal reflux disease; mRSS: modified Rodnan skin score; DLCO: diffusion capacity for carbon monoxide; FEV1: forced expiratory volume in 1 second; CRP: C-reactive protein, Hb: haemoglobin; PH : precapillary pulmonary hypertension *p-value less than 0.05
