# Supplementary material for: Predictors of lung function test severity and outcome in systemic sclerosis-associated interstitial lung disease
Source: PLoS One. 2017 Aug 1;12(8):e0181692. doi: 10.1371/journal.pone.0181692 (PMC5538660; doi:10.1371/journal.pone.0181692)
Supplement: S2 Table — (DOCX) [file pone.0181692.s004.docx]

S2 Table. Multivariate analysis of parameters associated with baseline value of FVC

| **Variable** |  | **coefficients** | **Standard error** | **p** |
| --- | --- | --- | --- | --- |
| Intercept |  | 79.0 |  |  |
| Baseline DLCO (% of predicted) | >80 | 15.8 | 6.0 | 0.001 |
| ILD extension according to Goh *et al*. | Limited | 12.5 | 6.1 | 0.046 |

FVC: forced vital capacity; DLCO: diffusion capacity for carbon monoxide; ILD: interstitial lung disease
