# Supplementary material for: Predictors of lung function test severity and outcome in systemic sclerosis-associated interstitial lung disease
Source: PLoS One. 2017 Aug 1;12(8):e0181692. doi: 10.1371/journal.pone.0181692 (PMC5538660; doi:10.1371/journal.pone.0181692)
Supplement: S3 Table — (DOCX) [file pone.0181692.s005.docx]

S3 Table. Decline in FVC, expressed as hazards ratio with 95% confidence intervals, in relation to baseline data (univariate analysis)

|  | HR | CI 95% | p |
| --- | --- | --- | --- |
| Age at diagnosis (years) | 0.59 | 0.26-1.30 | 0.19 |
| Sex | 1.54 | 0.68-3.52 | 0.20 |
| Disease duration since first Raynaud’s phenomenon | 0.61 | 0.28-1.33 | 0.21 |
| Disease duration since first non-Raynaud’s phenomenon | 1.24 | 0.56-2.74 | 0.29 |
| Ethnicity | 3.75 | 0.50-27.8 | 0.19 |
| Type of SSc | 1.68 | 0.77-3.66 | 0.19 |
| mRSS | 1.22 | 0.54-2.75 | 0.63 |
| Anticentromere antibodies | 0.57 | 0.08-4.30 | 0.59 |
| Anti-topoisomerase I | 0.90 | 0.41-1.97 | 0.79 |
| Dyspnoea (NYHA) | 1.94 | 0.84-4.51 | 0.12 |
| Respiratory symptoms leading to ILD diagnosis | 1.94 | 0.85-4.46 | 0.11 |
| GERD | 2.60 | 0.78-8.89 | 0.12 |
| Digital ulcers (presence or history) | 0.87 | 0.37-2.02 | 0.74 |
| Arthralgia | 0.69 | 0.29-1.60 | 0.38 |
| Synovitis | 0.53 | 0.53-1.84 | 0.31 |
| Baseline DLCO (%) | 0.26 | 0.08-0.89 | 0.03* |
| Baseline FVC (%) | 0.57 | 0.25-1.30 | 0.18 |
| CRP (mg/L) | 2.35 | 0.88-6.30 | 0.09 |
| Hb (g/dL) | 0.99 | 0.44-2.20 | 0.98 |
| Creatinine (µmol/l) | 0.56 | 0.24-1.26 | 0.16 |
| Extension of ILD (%) | 2.68 | 1.21-5.94 | 0.01* |
| Extension of reticulations  (%) | 1.30 | 0.60-2.83 | 0.50 |
| Proportion of ground-glass opacification (%) | 1.06 | 0.48-2.33 | 0.88 |
| Coarseness | 0.92 | 0.43-2.00 | 0.84 |
| Global score of bronchectasia | 0.78 | 0.36-1.73 | 0.55 |
| Emphysema extent (%) | 1.38 | 0.47-4.11 | 0.55 |
| ILD extension according to Goh *et al*. | 0.48 | 0.22-1.09 | 0.08 |
| ILD grade | 1.39 | 0.60-3.23 | 0.43 |
| PH by right heart catheterization at baseline or during follow up | 1.07 | 0.42-2.68 | 0.88 |
| Tricuspid regurgitation>2.8 ms^-1^ at baseline or during follow up | 1.41 | 0.65-3.10 | 0.38 |
| Immunosuppressants use during follow up | 3.46 | 1.55-7.70 | 0.002* |

FVC: forced vital capacity; SSc: systemic sclerosis; ILD: interstitial lung disease; GERD: gastro-oesophageal reflux disease; mRSS: modified Rodnan skin score; DLCO: diffusion capacity for carbon monoxide; FEV1: forced expiratory volume in 1 second; CRP: C-reactive protein, Hb: haemoglobin; PH : precapillary pulmonary hypertension *p-value less than 0.05 CI : confidence interval
