# Supplementary material for: Predictors of lung function test severity and outcome in systemic sclerosis-associated interstitial lung disease
Source: PLoS One. 2017 Aug 1;12(8):e0181692. doi: 10.1371/journal.pone.0181692 (PMC5538660; doi:10.1371/journal.pone.0181692)
Supplement: S4 Table — (DOCX) [file pone.0181692.s006.docx]

Supplemental Table 4 Multivariate analysis of parameters associated with baseline value of DLCO

| **Variable** | | **coefficients** | **Standard error** | **p** |
| --- | --- | --- | --- | --- |
| Intercept | | 53.1 |  |  |
| Respiratory symptoms leading to ILD diagnosis | Yes | -18.3 | 6.5 | 0.006 |
| Digital ulcers | Yes | 14.5 | 5.8 | 0.016 |
| Baseline FVC (%) | >80 | 17.6 | 6.4 | 0.008 |

ILD: interstitial lung disease; FVC: forced vital capacity
