# Supplementary material for: Predictors of lung function test severity and outcome in systemic sclerosis-associated interstitial lung disease
Source: PLoS One. 2017 Aug 1;12(8):e0181692. doi: 10.1371/journal.pone.0181692 (PMC5538660; doi:10.1371/journal.pone.0181692)
Supplement: S5 Table — (DOCX) [file pone.0181692.s007.docx]

Supplemental Table 5 Multivariate analysis of parameters associated with slope of DLCO

| **Variable** | **coefficients** | **p** |
| --- | --- | --- |
| Time | -0.7 |  |
| Presence of digital ulcers | -1.3 | 0.0141 |
| PH by right heart catheterization at baseline or during follow up | -1.2 | 0.0498 |

PH : precapillary pulmonary hypertension
